# Respiratory Shedding of Infectious SARS-CoV-2 Omicron XBB.1.41.1 Lineage among Captive White-Tailed Deer, Texas, USA

**DOI:** 10.3201/eid3102.241458

**Published:** 2025-02

**Authors:** Francisco C. Ferreira, Tahmina Pervin, Wendy W. Tang, Joseph A. Hediger, Logan F. Thomas, Walter E. Cook, Michael J. Cherry, Benjamin W. Neuman, Gabriel L. Hamer, Sarah A. Hamer

**Affiliations:** Texas A&M University, College Station, Texas, USA (F. Ferreira, T. Pervin, W.W. Tang, L.F. Thomas, W.E. Cook, B.W. Neuman, G.L. Hamer, S.A. Hamer); Texas A&M University—Kingsville, Kingsville, Texas, USA (J.A. Hediger, M.J. Cherry)

**Keywords:** SARS-CoV-2, COVID-19, severe acute respiratory syndrome coronavirus 2, coronavirus disease, respiratory infections, viruses, zoonoses, One Health, whole-genome sequencing, deer, *Odocoileus virginianus*, spillover, Texas, USA

## Abstract

White-tailed deer (*Odocoileus virginianus*) have high value for research, conservation, agriculture, and recreation and might be key SARS-CoV-2 reservoirs. In November 2023, we sampled 15 female deer in a captive facility in Texas, USA. All deer had neutralizing antibodies to SARS-CoV-2; respiratory swab samples from 11 deer were SARS-CoV-2–positive by quantitative reverse transcription PCR, and 1 deer also had a positive rectal swab sample. Six of the 11 respiratory swab samples yielded infectious virus; replication kinetics of most samples displayed lower growth 24–48 hours postinfection in vitro than Omicron lineages isolated from humans in Texas in the same period. Virus growth was similar between groups by 72 hours, suggesting no strong attenuation of deer-derived virus. All deer viruses clustered in XBB Omicron clade and demonstrated more mutations than expected compared with contemporaneous viruses in humans, suggesting that crossing the species barrier was accompanied by a high substitution rate.

The white-tailed deer (*Odocoileus virginianus*) (henceforth referred to as deer), a species native to North America, is highly susceptible to SARS-CoV-2 infection (*1*,*2*) and might serve as a viral reservoir because infected free-ranging deer can initiate onward intraspecies transmission (*3*,*4*). SARS-CoV-2 lineages have been detected in deer sampled up to 4–5 months after the same viral lineages were displaced from human populations by other lineages (*5*,*6*), suggesting sustained transmission within free-ranging animals. Transmission within deer can lead to a gradual accumulation of mutations in animal-adapted SARS-CoV-2 (*7*), which could alter infection kinetics or immune response when transmitted back into human populations (*8*). In addition, detections of deer-adapted virus infecting humans in Canada (*9*) and the United States (*3*) raise concerns about the emergence of sustained human–deer–human SARS-CoV-2 transmission cycles.

Captive animal herds with increased population densities and frequent human intervention might support transmission of zoonotic pathogens (*10*,*11*). Captive deer were also found to be naturally exposed to SARS-CoV-2, demonstrated by high rates of detection of viral RNA (*12*) and by high titers of neutralizing antibodies among deer (*13*). Managing highly susceptible animal species under human care creates opportunities for human-to-deer transmission and subsequent rapid dissemination within herds (*14*).

Data collected in 2012 estimated that ≈5,500 white-tailed deer breeding facilities exist in the United States (*15*). In the state of Texas, >1,000 existing facilities manage an average of 180–242 deer each (*16*,*17*). Having such large numbers of deer under human management might favor human-to-deer and deer-to-deer transmission events, leading to subsequent spread of deer-adapted viruses from farmed animals back into humans, which has been documented with farmed minks (*Neovison vison*) in Europe (*18*,*19*) and as a possibility in the United States (*20*).

Despite continued testing of deer for SARS-CoV-2 since the Omicron variant of concern emerged in the United States in November 2021, the detection rate of recent lineages in deer remains low (*3*,*21*). For example, only 2.5% (16 out of 648 genomes) of deer-derived sequences in GISAID (https://www.gisaid.org) as of August 14, 2024, belonged to the Omicron variant (GRA clade) (*22*). Those findings suggest deer are susceptible to new emerging lineages, yet the cause of a salient reduction in infection is unclear; reduced deer exposure to SARS-CoV-2, reduced virus fitness and detectability, protective immunity caused by exposure to pre-Omicron variants, reduced surveillance efforts, a lag between detection and reporting, or a combination of those factors could all play a role. Therefore, continued genomic surveillance of highly susceptible species remains critical to understand the current landscape of SARS-CoV-2 transmission and evolution in wildlife reservoirs.

We established an active surveillance program to determine SARS-CoV-2 circulation in captive deer in Texas to understand and mitigate transmission risk between humans and animals. In this study, we describe a SARS-CoV-2 outbreak in a captive deer-breeding facility in November 2023.

## Methods

### Cervid Facility

On November 15, 2023, we visited a private cervid facility in Milam County, Texas, as part of a larger project about SARS-CoV-2 surveillance in captive deer in the state. Deer were restricted to 2 pens separated by a fence and were able to have direct contact through the fence line. This facility falls into the category of outdoor ranch (*23*), although human–deer contact rates are high in our case because of veterinary care and animal husbandry.

### Deer Sampling

Fifteen apparently healthy female white-tailed deer were chemically immobilized for chronic wasting disease testing and endectocide treatment (unrelated to our research), at which time samples were collected for this SARS-CoV-2 investigation. We collected oral and nasal swab samples from deer using sterile polyester-tipped applicators with polystyrene handles (Puritan Medical Products, https://www.puritanmedproducts.com) and combined samples (henceforth referred to as respiratory swab samples) for each animal into a vial containing 3 mL of viral transport media (VTM) made following Centers for Disease Control and Prevention standard operating procedure number DSR-052-02. We collected rectal swab samples from the same animals into a separate vial with VTM. We collected blood (≈10 mL) from all animals through jugular venipuncture using sterile needles and syringes, transferred samples to tubes without anticoagulants, and aliquoted the obtained serum samples into microtubes. Our study followed relevant guidelines and regulations approved by the Texas A&M University Institutional Animal Care and Use Committee and Clinical Research Review Committee (2022-0001 CA).

### Molecular Detection and Whole-Genome Sequencing

We extracted total nucleic acid (TNA) from respiratory and rectal swab samples using the MagMax CORE Nucleic Acid Purification Kits on a 96-well Kingfisher Flex System (Thermo Fisher Scientific, https://www.thermofisher.com) following manufacturer instructions, using 200 μL of VTM from each sample and 90 μL of elution buffer. We added phosphate buffered saline 1× and a plasmid containing a portion of the RNA-dependent RNA polymerase SARS-CoV-2 gene to the TNA extraction plate as negative and positive extraction controls. We tested 5 μL of TNA for SARS-CoV-2 by quantitative reverse transcription PCR (qRT-PCR) targeting the RNA-dependent RNA polymerase (RdRp) gene of the virus following reagent concentrations as described in Corman et al. (*24*). We used molecular grade water as negative control and the same positive control described previously for qRT-PCR and yielded their expected results. We considered samples with cycle threshold (Ct) values <40 as positive.

We submitted all qRT-PCR–positive samples to whole-genome sequencing at the Texas A&M University Institute for Genome Sciences and Society Molecular Genomics Core. For each sample, 20 μL of TNA was submitted to library preparation using the xGen SARS-CoV-2 Amplicon Panel (Integrated DNA Technologies, https://www.idtdna.com). Sequencing was performed in an Illumina NovaSeq SP PE 2 × 150 flowcell version 1.5 (https://www.illumina.com) to generate an average of 3 million reads per sample.

### Bioinformatics

We processed raw reads for each sample through a pipeline developed by the Institute for Genome Sciences and Society Bioinformatics Core. Briefly, raw reads from the sequenced libraries were trimmed of low-quality bases and intact adaptor sequences using trim_galore version 0.6.10 (https://zenodo.org/records/5127899). We then mapped trimmed reads against the SARS-CoV-2 wild-type isolate genome assembly (National Center for Biotechnology Information RefSeq accession no. GCF_009858895.2) using the bwa aligner (*25*). Aligned reads for each sample then had variants called and filtered using BCFtools (*26*). Variant call files were then subset to further remove ambiguously called variants (defined as the ratio of an alternative variant called over the reference, <0.8) before International Union of Pure and Applied Chemistry (IUPAC) interrogation using a combination of Python and BCFtools. Finally, consensus sequences were called using BCFtools.

All SARS-CoV-2 genomes we obtained had >99.8% coverage. We interrogated positions with missing sequence data or mixed sequence data, indicated by IUPAC nucleotide uncertainty codes K, S, R, Y, M, W, or N in the assembled genome by BLAST+ 2.15.0 (https://blast.ncbi.nlm.nih.gov), to verify the presence of insertions/deletions and generate a majority-rule consensus sequence for each sample, which we used in subsequent phylogenetic analysis. We analyzed consensus sequences on Nextclade version 3.2.0 (https://clades.nextstrain.org) for clade and lineage assignment (*27*).

We investigated mutations, insertions, and deletions in the consensus sequences using NextClade and the GISAID database (*22*). Sequence differences are reported relative to the strain genetically closest to the deer-derived SARS-CoV-2 isolates (GISAID accession no. hCoV-19/USA/CA-HLX-STM-DTHMADVDZ/2023), which was collected from a human in California on November 11, 2023. We used UShER (https://genome.ucsc.edu/cgi-bin/hgPhyloPlace) (*28*) (assessed August 14, 2024) to determine the 10 available SARS-CoV-2 genomes most closely related to the sequences obtained in this study.

### Virus Isolation

We isolated SARS-CoV-2 in a Biosafety Level 3 laboratory at the Global Health Research Complex at Texas A&M University by passaging a mix of 100 μL of VTM with 900 μL of 1× Dulbecco modified Eagle medium through syringe filtration using an 0.2 micron pore size onto Vero E6-TMPRSS2-T2A-ACE2 (BEI Resources, https://www.beiresources.org) cells expressing both endogenous cercopithecine angiotensin-converting enzyme 2 (ACE2) and transmembrane serine protease 2 (TMPRSS2), as well as transgenic human ACE2 and TMPRSS2. We cultured these cells in Dulbecco modified Eagle medium supplemented with 10% fetal bovine serum, 1× antibiotic/antimycotic and puromycin dihydrochloride (10 µg/mL final concentration). We incubated cell plates at 37°C with 5% CO_2_ and collected the supernatants from samples exhibiting cytopathic effects within 24–72 hours for titration of infectivity. Those cytopathic effects are characterized by syncytium formation followed by rounding and detachment.

We performed titration of infectious virus by using the 50% tissue culture infectious dose (TCID_50_) method onto Vero E6-TMPRSS2-T2A-ACE2 cells. In brief, we prepared serial 3-fold dilutions of inocula in culture medium and then used them to inoculate monolayers of cells in tissue culture–treated 96-well plates. After 72 hours, we fixed the cells with phosphate buffered physiologic saline containing 25% final concentration of formalin and stained with crystal violet. We calculated titers of infectious virus using the Reed-Muench method (*29*).

### Virus Growth Curves

We used freshly titrated virus stocks with equal infectivity to inoculate onto Vero E6-TMPRSS2-T2A-ACE2 cells for 30 minutes, removed inoculum by 3 rinses with phosphate-buffered saline, and replaced it with culture medium. Through Global Health Research Complex at Texas A&M University, we obtained contemporaneous human clinical samples from nasal swab samples that were collected at various medical centers in southeastern Texas as part of an ongoing Texas Department of State Health Services virus surveillance project. We worked under approval from the Texas A&M University Institutional Review Board. The samples selected for use here included 1 isolate from each Pango lineage (*30*,*31*) obtained from patient clinical samples at our COVID-19 screening facility during October 1–November 30, 2023 (namely Pango lineages HV.1, HY.1, HK.11, HK.3, EG.10, EG.5, and JD.1) and were processed (isolated and titrated) in parallel with deer-isolated viruses. These human-derived isolates include all the distinct Pango lineages that were obtained 1 month before to 1 month after the deer-derived samples. In addition, the selected lineages represent the genotypes of 864 (38%) of 2,253 total sequenced SARS-CoV-2 complete genomes (excluding low-coverage and partial sequences) reported to GISAID from Texas during October 1–November 30, 2023. Performing experiments in parallel using human and deer samples enabled us to compare in vitro growth rates for both sample types. We collected small aliquots (100 μL) of medium at 8, 24, 48, and 72 hours after inoculation and submitted them to RNA extraction and Luna One-Step qRT-PCR (New England Biolabs, https://www.neb.com) targeting the nucleoprotein gene. We conducted a 1-way analysis of variance with Tukey correction for multiple comparisons to compare growth curves of human- and deer-derived isolates. Growth curve plots were generated using GraphPad version 10.0.0 (https://www.graphpad.com).

### Plaque Reduction Neutralization Tests

Serum samples from all deer were tested for the presence of neutralizing antibodies against SARS-CoV-2 (BEI Resources; isolate USAIL1/2020) through plaque reduction neutralization tests (PRNT) in Vero-CCL-81 (*13*). Aliquots of the samples were inactivated at 56°C for 30 minutes and screened at an initial dilution of 1:10. Samples that neutralized >90% of viral plaques, in comparison with the virus control, were further tested with serial 2-fold dilutions to determine the 90% endpoint titers (PRNT_90_).

## Results

### SARS-CoV-2 Detection and Serology in White-Tailed Deer

The respiratory swab samples of 11 (73.3%) of the 15 deer tested positive for SARS-CoV-2; of those SARS-CoV-2–positive deer, 1 (animal identification no. 231115-USDA-D89) also had a SARS-CoV-2–positive rectal swab sample, whereas all other rectal swab samples tested negative. The mean for the Ct values for respiratory samples was 27.93 (SD = 3.20, range 22.12–31.92) (Table).

**Table:** Quantitative reverse transcription PCR Ct values, virus isolation results, and PRNT_90_ results in study of respiratory shedding of infectious SARS-CoV_2 Omicron XBB 1.41.1 lineage among captive white-tailed deer (*Odocoileus virginianus*), Texas, USA, November 2023*

Animal identification no.	Ct value for respiratory swab samples	Ct value for rectal swab samples	Virus isolation from respiratory swab samples	PRNT_90_ endpoint titer
231115-USDA-D87	28.04	NA	Negative	1:80
231115-USDA-D88	29.02	33.44	Negative†	1:320
231115-USDA-D89	31.62	NA	Negative	1:80
231115-USDA-D90	28.30	NA	Negative	1:80
231115-USDA-D91	NA	NA	NA	1:640
231115-USDA-D92	28.66	NA	Positive	1:160
231115-USDA-D93	28.11	NA	Positive	1:20
231115-USDA-D94	24.73	NA	Positive	1:40
231115-USDA-D95	22.12	NA	Positive	1:40
231115-USDA-D96	23.78	NA	Positive	1:10
231115-USDA-D97	NA	NA	NA	1:160
231115-USDA-D98	NA	NA	NA	1:20
231115-USDA-D99	NA	NA	NA	1:20
231115-USDA-D100	31.92	NA	Positive	1:10
231115-USDA-D101	31.07	NA	Negative	1:160

*Ct, cycle threshold; NA, not applicable; PRNT_90_, 90% plaque reduction neutralization test. †Neither respiratory nor rectal swab samples yielded infectious virus.

All 15 deer had neutralizing antibodies against SARS-CoV-2 as demonstrated by PRNT. PRNT_90_ endpoint titers varied from 1:10 to 1:320 in qRT-PCR–positive animals (mean 91, SD 93) and ranged from 1:20 to 1:640 in qRT-PCR–negative animals (mean 210, SD 294) (Table).

### Virus Isolation

**Figure 1:**
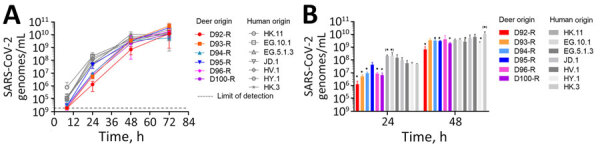
Multistep growth characteristics of contemporaneous strains isolated from captive white-tailed deer and humans in study of respiratory shedding of infectious SARS-CoV-2 Omicron XBB 1.41.1 lineage among captive white-tailed deer, Texas, USA, November 2023. A) Vero E6-TMPRSS2-T2A-ACE2 cells were inoculated with SARS-CoV-2 recovered from deer (D92-R to D100-R) or from human clinical nasopharyngeal samples (EG.10.1, EG.5.1.3, HK.11, JD.1, HV.1, HY.1, HK.3) at a multiplicity of infection of 0.002. Samples of the supernatant were collected and titrated by 1-step quantitative PCR. Lower limit of detection is indicated. B) Averaged data for the 24-hour and 48-hour timepoints. Statistically significant differences after 1-way analysis of variance with Tukey correction are indicated by asterisks for values significantly lower than aggregated human samples and asterisks in parentheses for values significantly higher than aggregated human samples. Error bars indicate SDs calculated from 3 replicates. We added “-R” to the name of each animal identification number to indicate that samples used in these experiments were recovered from the initial virus isolation step.

To assess whether deer-derived SARS-CoV-2 was able to efficiently use human ACE2 and TMPRSS2 for cell entry, we inoculated Vero E6-TMPRSS2-T2A-ACE2 cells with all 12 qRT-PCR–positive respiratory and rectal swab samples. Six respiratory samples (D92, D93, D94, D95, D96, D100) (Figure 1) produced cytopathic effects characteristic of SARS-CoV-2 on this cell line (Table). Each of those viral isolates was designated as recovered to differentiate the isolates from the potentially broader SARS-CoV-2 populations present in the original samples from infected animals (Figure 1). We grew and titrated stocks of each isolate by the TCID_50_ method for further study.

To compare growth rates of viruses isolated from deer with strains that were circulating in humans in Texas at the same time, we performed multistep growth curves, starting at low multiplicity of infection (0.002). We observed that 5 of 6 deer-derived viruses showed lower growth rates than did human-derived isolates at 24 hours, and 4 of 6 deer-derived viruses showed lower growth rates than did human-derived isolates at 48 hours (p<0.05) (Figure 1, panel B). We noted no differences in growth at 8 hours or 72 hours (Figure 1, panel A). Those data indicated that deer-derived SARS-CoV-2 isolates were able to efficiently grow in cells expressing human ACE2 and TMPRSS2, with a delay in early growth and no difference in burst size compared with contemporaneous SARS-CoV-2 isolated from humans.

### SARS-CoV-2 Sequencing

**Figure 2:**
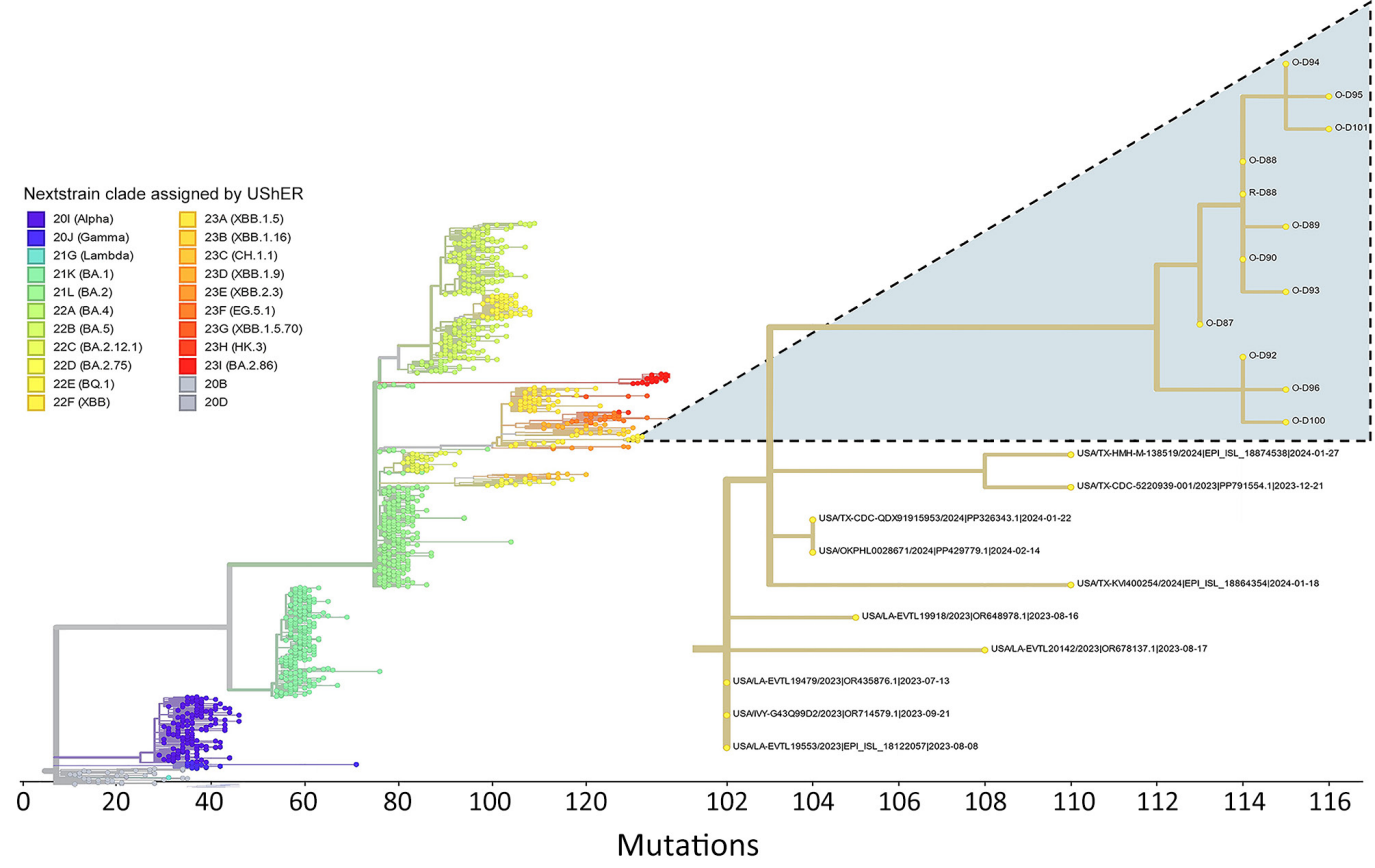
Phylogenetic context of SARS-CoV-2 from captive white-tailed deer in study of respiratory shedding of infectious SARS-CoV-2 Omicron XBB 1.41.1 lineage, Texas, USA, November 2023. Genome sequences from deer were compared in the main phylogenetic tree with genomes representative of the main virus Nexstrain clades as assigned by UShER (https://genome.ucsc.edu/cgi-bin/hgPhyloPlace) (28). The secondary tree at right details the 22F (XBB) clade and displays the placement of genomes obtained in our study (highlighted clade; O indicates oral/nasal swab, R indicates rectal swab) in relation to 10 of the most closely related samples deposited in GISAID (https://www.gisaid.org) as of August 14, 2024.

We obtained complete (>99.8%) SARS-CoV-2 genomes from all 12 qRT-PCR–positive samples (11 respiratory samples and 1 rectal swab sample) belonging to 11 animals. Analysis by Nextclade revealed that all viruses fell within the XBB clade (XBB.1.41.1 lineage assigned by Pangolin version 4.3, also known as JC.5). Viruses most closely related to the ones sequenced from deer were collected from humans in California on November 11, 2023 (GISAID accession no. hCoV-19/USA/CA-HLX-STM-DTHMADVDZ/2023); from Texas on December 19, 2023; and from Texas in January 2024 (Figure 2). Those sequences from humans were obtained from different GISAID submitters and had identities ranging from 95.5% (1,352-base difference) to 99.9% (21-nt difference) in relation to the sequences we obtained from deer. No SARS-CoV-2 detected in deer in other regions of North America clustered together with the genomes obtained here.

Our deer-derived sequences had 15 nt substitutions not found in the closest human-derived isolates and up to 11 substitutions shared by some or none of the other deer-derived sequences. Compared with those closely related human samples, the viruses detected in deer did not have unique mutations in the spike protein. However, the viruses detected from humans in Lubbock County, Texas, and in Oklahoma had amino acid changes (N74S [Lubbock County] and Q146H [Oklahoma]) not present in the deer samples when using the lineage XBB as a reference.

## Discussion

All 15 deer sampled in a captive facility in Texas had evidence of exposure to SARS-CoV-2, and 11 (73%) deer were shedding viral RNA, showing a widespread virus dissemination among the animals. In addition, whole-genome sequencing revealed that recent Omicron lineages continue to be detected within deer in North America. Of the qRT-PCR–positive deer assessed, 6 had infectious virus in their upper respiratory tract. Although viruses isolated from most deer showed slower growth at 24 hours and 48 hours after inoculation in vitro than did contemporaneous human-isolated viruses, the number of viral copies at 72 hours was similar between groups, showing no attenuation in deer-adapted viruses. The XBB lineage detected in our study was circulating at low frequency in human populations in the United States; cumulative prevalence was <0.5% during May 2023–January 2024 (*32*), concurrent with the period in which deer were sampled for this study.

Human-derived SARS-CoV-2 have 30–32 substitutions/genome/year (≈2.5/month; https://nextstrain.org/ncov/gisaid/global/6m?l = clock). The human strain hCoV-19/USA/CA-HLX-STM-DTHMADVDZ/2023 and our deer samples were collected 7 days apart and displayed 15–22 nt substitutions among them, suggesting that crossing the species barrier from human to captive deer was accompanied by a high substitution rate. However, determining that pace is difficult without knowing when the initial spillover into this population occurred. Possible explanations for the proposed burst of evolution include bottlenecking and strong activation of mutagenic intracellular factors, such as RNA editing enzymes (ADAR1) and apolipoprotein B mRNA editing enzyme, catalytic polypeptide-like (APOBECs) in deer (*9*). For instance, APOBECs play a key role during natural infections, and the catalytic subunit 3H was shown to display stronger upregulation in infected deer than in humans with natural infections (*33*). Furthermore, the comparable to slightly attenuated growth of deer and human isolates suggests that deer-to-human transmission, and concomitant bursts of evolution within deer, have the potential to produce variants that might not display substantial loss of adaptation for infection and development in humans. This finding suggests that Omicron SARS-CoV-2 lineages circulating in deer might be efficiently transmitted back to humans.

Further, the role of other wild or domestic animals in generating, harboring, or transmitting mutated virus at such deer facilities is unknown. The XBB clade has been detected in multiple wildlife species in areas within a rural-to-urban gradient; >5 transmission events from human to animals have occurred (*34*). Because wildlife species susceptible to SARS-CoV-2 share habitat with captive deer (*34*,*35*), our results suggest that infectious deer have the potential to infect and be infected by commingling species. Assessments of wildlife, livestock, and humans that commingle with infected captive deer are a high priority for testing for cross-species transmission events.

Free-ranging deer are predicted to be at higher risk for exposure to SARS-CoV-2 when sharing fence line with captive populations (*23*), and the high frequency of infectious deer we detected emphasizes that transmission from captive to wild deer should be empirically tested. In addition, fence line transmission could explain, at least partially, the widespread sustained SARS-CoV-2 transmission among wild deer in North America (*36*).

Some wildlife species found to be shedding RNA of XBB lineages under natural conditions (e.g., raccoons [*Procyon lotor*] and cottontail rabbits [*Sylvilagus floridanus*]) (*34*) did not show clinical signs of infection, nor did they shed viral RNA when experimentally infected with early, pre-Omicron lineages (*37*,*38*). Those studies together provide strong evidence that, as new lineages emerge, the breadth of competent hosts for the virus changes, highlighting the importance of continued surveillance to assess risks of establishment of transmission cycles of SARS-CoV-2 among wildlife species.

Infectious virus can be isolated from nasal swab samples up to 6 days after infection in deer, whereas viral RNA can be detected in respiratory swab samples up to 22 days after direct experimental infection and up to 21 days after contact with infected deer (*1*,*2*). Those studies, which used pre-Omicron lineages, revealed efficient horizontal transmission among deer. Of the qRT-PCR–positive deer from our study, 6 of 11 had infectious virus, suggesting they became infected within ≈6 days before our sampling, although that timeframe might vary under natural conditions compared with laboratory-based experiments. Nevertheless, our results show that more recent lineages are also efficiently and quickly transmitted among deer. However, controlled laboratory experiments are needed to confirm the duration of infectious virus shedding during infections with more recent SARS-CoV-2 lineages in deer.

Our study demonstrates the continued and efficient spread of SARS-CoV-2 among deer as new virus lineages emerge and infect nonhuman species; an XBB lineage concurrently circulating in human populations spread rapidly within deer. These findings corroborate epidemiologic models predicting that deer facilities favor high virus dissemination within captive populations (*23*), and transmission potential might vary across the spectrum of scenarios in which deer and humans interact (e.g., deer ranches, wildlife sanctuaries, safaris, zoos, outdoor recreation with free-ranging animals). Our study cannot determine how SARS-CoV-2 entered the target deer population. This knowledge gap needs to be addressed to mitigate transmission risks among humans, deer, and other commingling species. Given the uncertain public health and animal health implications of viral maintenance within captive deer, agricultural biosecurity practices could be useful in reducing the possibility of establishing long-term animal reservoirs for the virus. The mid- to long-term evolutionary consequences for SARS-CoV-2 circulating in nonhuman populations is unknown, and captive facilities might provide opportunities for sustained transmission at this human–deer–wildlife interface. 
